# Supplementary material for: Laminin Receptor-Mediated Nanoparticle Uptake by Tumor Cells: Interplay of Epigallocatechin Gallate and Magnetic Force at Nano–Bio Interface
Source: Pharmaceutics. 2022 Jul 22;14(8):1523. doi: 10.3390/pharmaceutics14081523 (PMC9330565; doi:10.3390/pharmaceutics14081523)
Supplement: Supplementary file 1 [file pharmaceutics-14-01523-s001.zip › pharmaceutics-1790631-supplementary.pdf]

pharmaceutics

Supplementary Materials: Laminin Receptor-Mediated Nanoparticle Uptake by Tumor Cells: Chemical and Magnetic Interplay at Nano-Bio Interface

Sheng-Chieh Hsu, Nian-Ping Wu, Yi-Ching Lu and Yunn-Hwa Ma

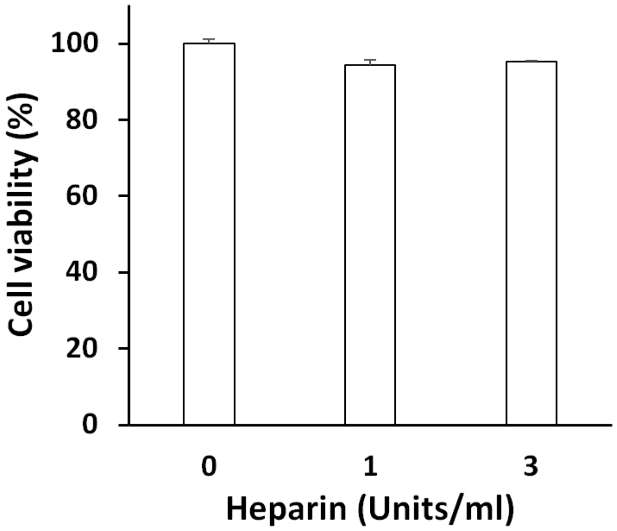

Figure S1. Heparin did not exhibit an effect on LN-229 cell viability. After 24 h treatment with heparin (1 and 3 Unit/mL), cell viability was determined by CCK-8 assay.

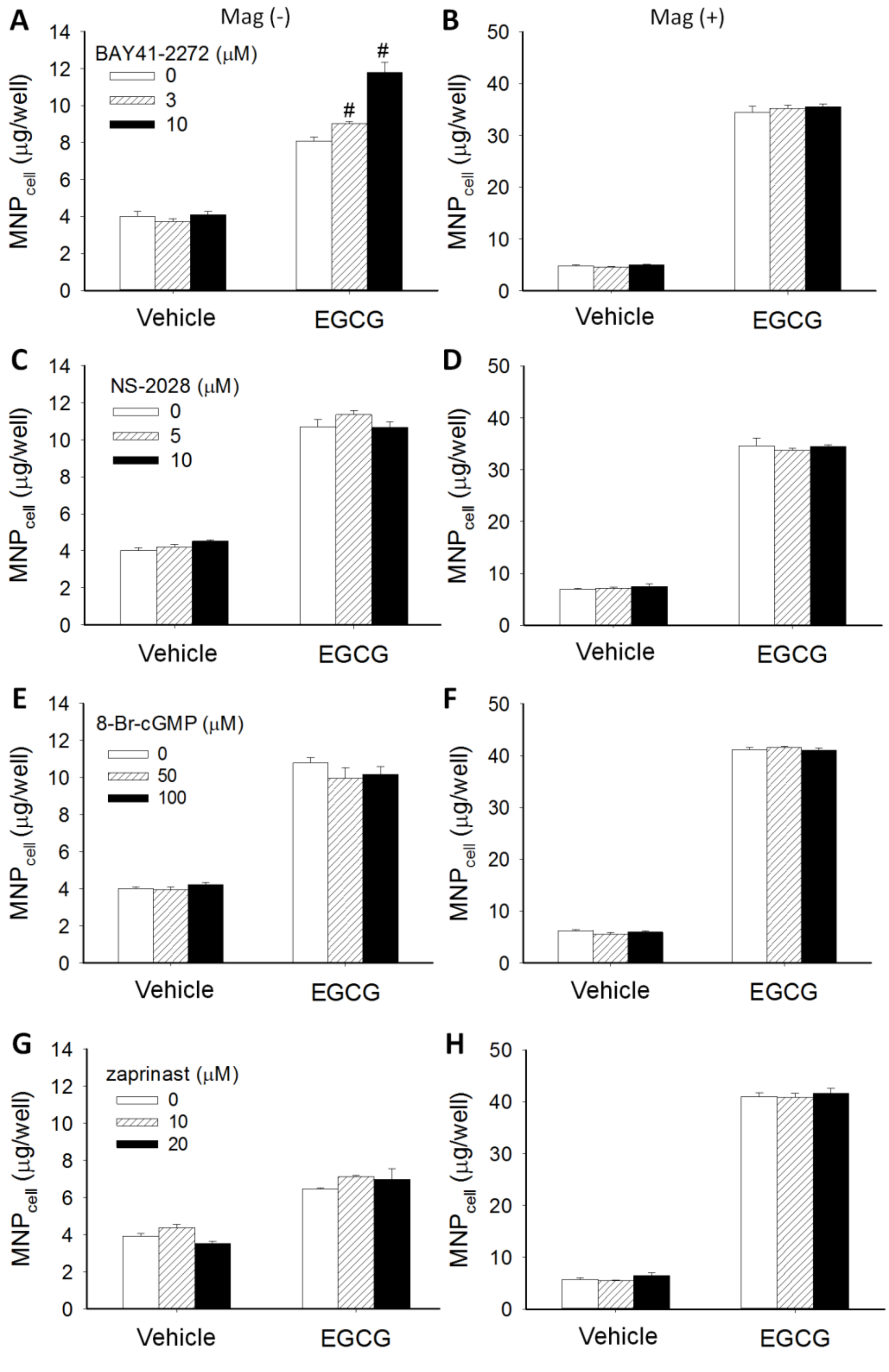

Figure S2. Effects of pharmacological agents that modulate cGMP levels on EGCG-induced MNP uptake. Cell-associated MNPs (MNPcell) were determined in response to BAY41-2272 (an activator of soluble guanylate cyclase, 3 or 10 μM; **A**, **B**), NS-2028 (an inhibitor of soluble guanylate cyclase, 5 or 10 μM; **C**, **D**), 8-Br-cGMP (50 or 100 μM; **E**, **F**) or zaprinast (a PDE inhibitor, 10 or 20 μM; **G**, **H**). LN-229 cells exposed to MNPs (CMX-MNP; 50 μg/well) and EGCG (10 μM) in the absence (left panels; Mag–) or presence (right panels; Mag+) of magnet for 2 h. Values are mean ± SEM (n = 4); the results are representative of 3 experiments using different batch of cells. #, $p < 0.05$ compared with corresponding group without BAY41-2272.

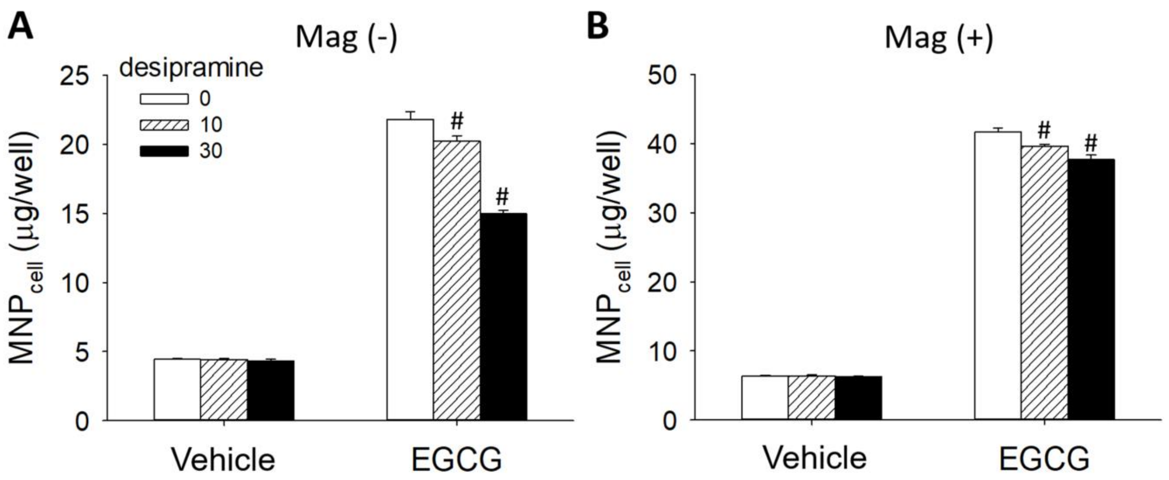

Figure S3. Acid sphingomyelinase inhibitor attenuated EGCG-induced MNP uptake. After 30-min pretreatment with desipramine (an acid sphingomyelinase inhibitor; 10 or 30 μM), LN-229 cells were incubated with MNPs (dextran-MNP: 50 μg/well) and EGCG (10 μM) in the absence (**A**) or presence (**B**) of the magnet for 6 h. Values are mean ± SEM (n = 4); the results are representative of 4 experiments using different batch of cells. #, $p < 0.05$ compared with corresponding group without desipramine.

supplementary.avi

Video S1: EGCG enhanced MNP aggregation and internalization by glioma cells.
